# Early Rehabilitation Program and Vitamin D Supplementation Improves Sensitivity of Balance and the Postural Control in Patients after Posterior Lumbar Interbody Fusion: A Randomized Trial

**DOI:** 10.3390/nu11092202

**Published:** 2019-09-12

**Authors:** Wojciech Skrobot, Ewelina Liedtke, Katarzyna Krasowska, Katarzyna P. Dzik, Damian J. Flis, Anna Samoraj-Dereszkiewicz, Witold Libionka, Jakub Kortas, Wojciech Kloc, Jedrzej Antosiewicz, Jan J. Kaczor

**Affiliations:** 1 Department of Kinesiology, Faculty of Rehabilitation and Kinesiology, Gdansk University of Physical Education and Sport, 80-336 Gdansk, Poland; ewelinaliedtke@gmail.com (E.L.); katarzyna.krasowska@awf.gda.pl (K.K.); samoraj.anna@gmail.com (A.S.-D.); 2 Faculty of Physical Education, Gdansk University of Physical Education and Sport, 80-336 Gdansk, Poland; kasi.dzik@gmail.com (K.P.D.); jakubantonikortas@gmail.com (J.K.);; 3 Department of Neurobiology of Muscle, Faculty of Rehabilitation and Kinesiology, Gdansk University of Physical Education and Sport, 80-336 Gdansk, Poland; wlibionka@yahoo.com (W.L.); jacek.kaczor@awf.gda.pl (J.J.K.); 4 Department of Bioenergetics and Nutrition, Faculty of Rehabilitation and Kinesiology, Gdansk University of Physical Education and Sport, 80-336 Gdansk, Poland; damianflis19@wp.pl; 5 Department of Neurosurgery, Copernicus Hospital in Gdansk, 80-336 Gdansk, Poland; wk56rak@gmail.com; 6 Department of Health Promotion, Faculty of Tourism and Recreation, Gdansk University of Physical Education and Sport, 80-336 Gdansk, Poland; 7 Department of Neurology and Neurosurgery, University of Warmia and Mazury in Olsztyn, 10-719 Olsztyn, Poland; 8 Department of Bioenergetics and Physiology of Exercise, Faculty of Health Sciences, Medical University of Gdansk, 80-336 Gdansk, Poland

**Keywords:** supplementation, vitamin D, rehabilitation exercise, lumbar spinal fusion, proprioception

## Abstract

Background: The introduction of early rehabilitation exercise is the foundation of treatment post-Posterior lumbar interbody fusion (PLIF) surgery, and the search for additional sources of reinforcement physiotherapy seems to be very important. Methods: The patients were randomly divided into the vitamin D3 (*n* = 15; D3) supplemented group and received 3200 IU per day for five weeks before surgery and the placebo group (*n* = 18; Pl) received vegetable oil during the same time. The patients began the supervisor rehabilitation program four weeks after surgery. Results: The limits of stability (LOS) were significantly improved in the D3 group after 5 and 14 weeks (*p* < 0.05), while in the Pl group, progress was only observed after 14 weeks (*p* < 0.05). The LOS were also higher in the D3 group than in the Pl group after five weeks of supervised rehabilitation (*p* < 0.05). In the postural stability (PST) test, significant progress was observed in the D3 group after 14 weeks (*p* < 0.02). In addition, neither rehabilitation nor supplementation had significant effects on the risk of falls (RFT). Conclusions: Vitamin D supplementation seems to ameliorate the effects of an early postoperative rehabilitation program implemented four weeks after posterior lumbar interbody fusion. Early physiotherapy treatment after PLIF surgery combined with vitamin D supplementation appears to be a very important combination with regard to the patients’ recovery process.

## 1. Introduction

Proper and rapid bony fusion of the stabilized spinal segment in the patients who have undergone a posterior lumbar interbody fusion (PLIF) procedure is of the utmost importance regarding the clinical outcome of the surgery. However, although there is no standard rehabilitation protocol for the patients after PLIF surgery, there is evidence of the positive effects of rehabilitation exercise [1]. Early rehabilitation is particularly important in this group of patients as it has been reported that muscle damage and denervation is greater after PLIF, and a period of postoperative immobilization is longer when compared to a simple lumbar discectomy [2,3]. Movement is considered to be essential to activate and strengthen supporting muscles, which are responsible for maintaining spinal stability. In addition, appropriate exercises affect the control of stability and flexibility, as well as stimulate blood flow to the wound-healing site. During the early postoperative phase, therapeutic exercise has been suggested to keep the lumbar spine in a neutral position to minimize the strain on the fused segment. Moreover, therapeutic exercise programs are also required to stabilize the lumbar spine and lumbo-pelvic complex [1,4]. Thus, rehabilitation programs for patients are mainly focused on improving the weakness of muscles that occurs in the segments with a disc injury [4]. Lumbar stabilization exercise has been proven to eliminate local weakness and ameliorate movement control after any spinal surgical procedures [4].

However, there are still some controversies regarding the initiation of back muscle exercises after spinal fusion surgery. Some studies have demonstrated that rehabilitation exercises initiated within three months after surgery [5] or six months after lumbar spinal fusion are safe and efficacious [6]. Certainly, in addition to exercise, there are other factors that can increase skeletal muscle strength, reduce the local inflammation process, improve proprioception, and therefore, positively influence the recovery process. In the authors’ opinion, one of the elements of complete rehabilitation factors is vitamin D. It has been demonstrated that vitamin D influences the expression of approximately 900 genes [5]. Moreover, vitamin D deficiency has been associated with muscle weakness, inflammation, and impaired proprioception [6,7,8,9,10]. Moreover, it has been associated with diminished citrate synthase (CS) activity and the reduced protein content of PGC-1α in the skeletal muscle of the patients with chronic low back pain [9]. In addition, the attenuated level of blood C-reactive protein (CRP) and interleukin 6 (IL-6) have been observed in the patients supplemented with vitamin D3 [11]. Recently, Alamdari and coworkers showed that there was an inverse correlation between nerve conduction velocity and concentration of 25(OH)D3 in diabetic patients [12]. An increasing number of studies have indicated that vitamin D deficiency is a common problem among both young and elderly people. For example, a large number of orthopedic patients have a vitamin D deficiency, which is associated with longer hospitalization [13]. In another study, it has been demonstrated that 84.7% of the patients with osteoarthritis of the knee or hip had a vitamin D deficiency [14]. 

Most of the vitamin D comes from skin synthesis, thus low sun exposure and the application of sunscreen seems to be the main reason for vitamin D deficiency. Vitamin D is synthesized in the skin from 7-dehydrocholesterol, the concentration of which is significantly decreased in elderly people. Therefore, it has been suggested that the lack of vitamin D precursor might be another significant factor of vitamin D deficiency in elderly people [15]. Moreover, dietary sources of the vitamin are scarce, and the consumption of fish and fish fat is insufficient in many countries. Together, vitamin D deficiency is common, especially among people living in northern countries, including Poland [16]. The exploration of new ways to support rehabilitation programs following lumbar fusion surgery is still necessary. Thus, the main objective of this work was to check whether vitamin D supplementation strengthens the influence of a rehabilitation program on balance after PLIF surgery. This study hypothesized that the effects of an early rehabilitation program on postural control can be improved when combined with vitamin D supplementation in the patients after posterior lumbar interbody fusion.

## 2. Materials and Methods

The purpose of the current study was to evaluate the impact of an early rehabilitation program and vitamin D supplementation on the sensitivity of balance and postural control in the patients after posterior lumbar interbody fusion.

### 2.1. Design

**Figure 1:**
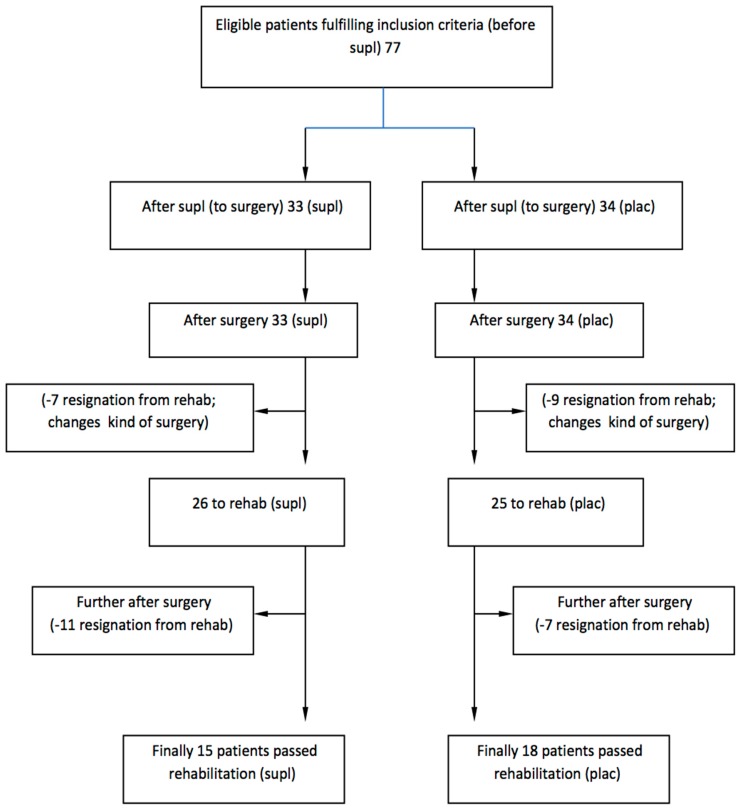
Consort flow digram of the study.

The study was a double-blinded, randomized controlled trial (Figure 1).

### 2.2. Participants, Therapists, Centers

All the patients underwent a neurological and magnetic resonance imaging (MRI) examination. The patients were classified with an operation protocol by a neurosurgeon.

The inclusion criteria for participation in the study and PLIF surgery were: Aged between 20 and 70 years; symptomatic (back pain and/or sciatica exceeding 12 months) spinal stenosis; spondylosis; degenerative or isthmic spondylolisthesis, or degenerative disc disease with segmental instability for which conservative treatment had failed.

The exclusion criteria included previous lumbar fusion, ankylosing spondylitis, rheumatoid arthritis, and the worsening of existing or the appearance of new symptoms.

### 2.3. Intervention

The patients were randomly divided into vitamin-D-supplemented (D3) and placebo (Pl) groups. The D3 group was supplemented with vitamin D (Vigantol, Merck) of 3200 IU/day, for five weeks before surgery and the placebo group (Pl) group obtained the same volume of vegetable oil. Trained physiotherapists performed the rehabilitation procedure for all the patients in both groups. Another physiotherapist conducted the functional tests. All the patients gave full informed consent to participate in the study and could leave the study at any time.

The patients started the supervised rehabilitation protocol four weeks after surgery. The rehabilitation took place at the Gdansk University of Physical Education and Sport. The study was conducted during the period between October and April when UV-stimulated vitamin D synthesis is close to zero.

The study was approved by the local institutional Bioethical Committee in Gdansk (No. NKBBN/120/2012), conformed to the Declaration of Helsinki guidelines, and was registered as a clinical trial (NCT03417700).

### 2.4. Outcome Measures

The Biodex Balance System (BBS) was used to assess the control of balance through three tests: The postural stability test (PST); the limits of stability test (LOS); and the risk of fall test (RFT). The PST was performed four times: Before supplementation with vitamin D3 or placebo (week 0); five weeks after supplementation, 2 ± 1 days before surgery (week 5); four weeks after surgery (week 9); and five weeks after supervised rehabilitation (week 14). The LOS and RFT tests were performed at weeks 0, 5, and 14 due to a very short postoperative period and adhesion in the segment. Prior to testing, the participants underwent a familiarization session. The participants were asked to step on a platform in a bipedal stance with bare feet and open eyes looking forward to the BSS monitor to control the cursor, while their hands hung by their sides (hand support was not permitted). They were asked to stand straight, not to change their feet position, and only sway their body when necessary.

#### 2.4.1. Limits of Stability Test (LOS)

During the test, the platform was stable. The LOS test is defined as the maximum angle a body can achieve from vertical without losing one’s balance. When the LOS is exceeded, a fall, stumble, or step will ensue. The BBS provides scores for all eight directions as well as an overall score. The higher scores indicate a better performance. The subjects performed three trials of the LOS test, which involved shifting their body weight while standing on a platform to move a cursor on the screen from a center target to a peripheral blinking target [17].

#### 2.4.2. Postural Stability Test (PST)

The BBS allowed an objective evaluation of postural stability through three indexes: The overall stability index (OSI), the anterior-posterior stability index (APSI), and the medial-lateral stability index (MLSI). During the tests, the platform was stable. This test consisted of three trials, each with a duration of 10 s. These indexes represent the fluctuations around a zero point established prior to testing when the platform is stable [18].

The PST consisted of three measurements. Each measurement was taken on a static platform at intervals of 20 s of testing and a 10 s break. The higher scores indicate a greater amount of postural instability. The participants were asked to step on a platform in a bipedal stance with bare feet and open eyes looking forward to the BSS monitor, while their hands hung by their sides. They were asked to stand straight, not to change their feet position, and only sway their body when it was necessary.

#### 2.4.3. Risk of Fall Test (RFT)

The assessment of the dynamic bilateral stance was conducted on an unstable platform, where levels 6 to 2 were used. The test consisted of three measurements at intervals of 20 s of testing, and a break of 10 s. Prior to testing, the test procedure was explained. In this test, only the OSI was performed. This index was calculated through the degree of oscillation of the platform, where lower scores suggested better stability of the body.

#### 2.4.4. Rehabilitation Protocol

Before the operation, the patients were informed and instructed on how to move ergonomically both in the hospital and at home after the operation treatment.

During the first weeks after surgery, the patients were encouraged to initiate spontaneous activity, consisting mainly of walks with an extended distance with some self-control and without pain.

The patients initiated an individually supervised rehabilitation protocol four weeks after PLIF surgery that lasted for five weeks (three times a week for one hour). All the participants performed the same procedures, and the difficulty of the tasks increased with every week of the rehabilitation protocol (Table 1).

Physiotherapists were trained in the rehabilitation protocol before the start of the exercises and informed the coordinator about any worsening of the patient’s symptoms. The rehabilitation exercises were conducted without pain.

During the early postoperative phase, strengthening exercises should be performed while keeping the lumbar spine in a neutral position to minimize strain on the fused/adjacent segment. This procedure was used to avoid breakage of the fusion device or dislocation of the pedicle screws. In functional neutral spine control exercises, a destabilizing force acts on the trunk through the loading of the extremities, therefore, proper recruitment of the trunk muscles is required to stabilize the lumbar spine and lumbo-pelvic complex [1].

### 2.5. Data Analysis

A statistical analysis was performed using Statistica 12.0 software (Statsoft, Tulsa, OK, USA). All values were expressed as the mean ± standard deviation (SD). The Shapiro–Wilk test was applied to assess the homogeneity of dispersion from the normal distribution. The Brown–Forsythe test was used to evaluate the homogeneity of variance. For homogenous results, an analysis of variance (ANOVA) for repeated measures and post-hoc least significant difference (LSD) test for unequal sample sizes were performed to identify significantly different results. For heterogeneous results, an ANOVA Friedman’s test and the right post-hoc test were applied. The significance level was set at *p* < 0.05.

## 3. Results

The serum 25(OH)D3 concentration in the PLIF patients was similar in both the placebo and D3 group. After five weeks of supplementation, 25(OH)D3 was significantly higher in the D3 group when compared to the PL group (Table 2, *p* < 0.005).

**Figure 2:**
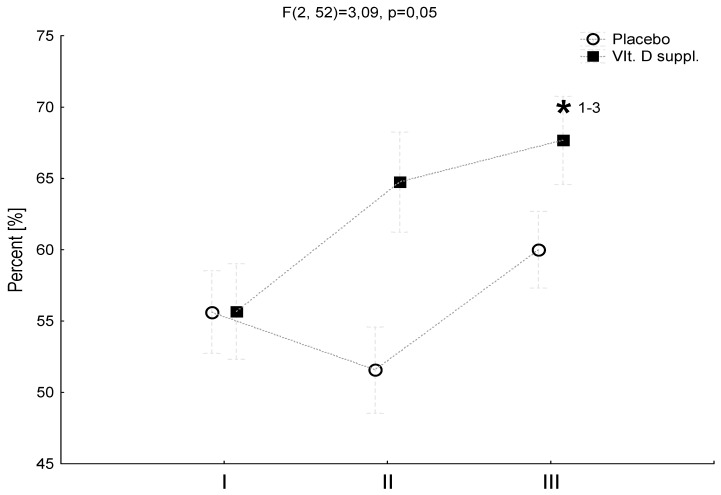
The effect of vitamin D supplementation on the limits of stability test (LOS) in the patients before and after surgery, and the rehabilitation program. The placebo group (Pl) group (*n* = 15) and D3 group (*n* = 11). The columns, mean; bars, SD. * *P* < 0.05, significantly different when compared with before supplementation. The tests were performed: I = before supplementation; II = after five weeks of supplementation; III = after four weeks home based rehabilitation and five weeks of supervised rehabilitation. LOS = limits of stability test.

Five weeks after vitamin D supplementation prior to surgery, a significant improvement in the LOS was observed in the D3 group while in the Pl group, non-significant differences occurred (Figure 2).

At the end of the rehabilitation program (week 14), the LOS significantly increased when compared to the values obtained before surgery in both the Pl and D3 groups. Interestingly, the LOS increase in the D3 group was higher when compared to the Pl group at the end of the rehabilitation program (Figure 2).

Furthermore, there was a tendency to improve the scores of PST in the D3 group (Table 2).

The PST index was significantly lower after five weeks of vitamin D supplementation in the D3 group and did not change in the Pl group. Five weeks of supervised rehabilitation significantly improved all three indexes (OSI, APSI, MLSI) of stability, but only in the D3 group (Table 3).

The RFT did not alter in both groups after five weeks of vitamin D supplementation (Table 2 and Table 3) and there were no significant changes in the RFT after an early rehabilitation program in both groups of patients (Table 3).

## 4. Discussion

The most important finding of this study was that the patients after PLIF surgery that had an early rehabilitation program and were supplemented with vitamin D3 appeared to have better short-term outcomes of the objective parameters, like body proprioception control when compared to the patients who only underwent the rehabilitation program. In addition, the pre-operative vitamin D supplementation exerted significant positive effects.

The LOS significantly increased after vitamin D supplementation alone and further improved after the combined home-based and supervised rehabilitation program (week 14). These data were in agreement with a previously published study that demonstrated that post-operative outcomes following total knee arthroplasty were affected by the patients’ pre-operative vitamin D status [19]. Several studies have shown an association between vitamin D deficiency and spine and joint morbidities [13,20]. However, to the best of the authors’ knowledge, this is the first study where the combined effects of vitamin D supplementation and a rehabilitation program were evaluated.

These data clearly show that postural control was considerably improved in both groups and that vitamin D had positive effects. Vitamin D deficiency is quite common among people living in northern countries [21,22]. For example, it has been shown that 92% of elderly women in Poland have a 25(OH)D3 concentration below 50, and 25% below 25 nmol/L during the winter season [16]. In addition, ageing, low physical activity and obesity are risk factors of vitamin D deficiency [15,23,24]. There is still some controversy about the optimal concentration of vitamin D. It has been reported that a concentration of 50 nmol/L is sufficient to completely protect against rickets. However, a much higher concentration is needed to reduce the risk of cancer and other morbidities [25]. In our study, we observed some beneficial effects of vitamin D supplementation even in patients whose baseline concentration of 25(OH)D3 was above 50 nmol/L. These data indicate that possibly higher concentrations of 25(OH)D3 are optimal to support the rehabilitation of patients after PLIF surgery.

The data show the positive influence of vitamin D on postural control; this observation is in agreement with a previous report that showed abnormal limb proprioception and body sways in subjects with low concentrations of 25(OH)D3 [6,26].

The limits of the stability test, which assesses balance in the dynamic state by immediately tracking the change in velocity and the position of center of mass, confirmed the beneficial effects of vitamin D supplementation and the rehabilitation program. Moreover, the LOS is an indicator of proprioception improvement and dynamic balance in postural control [27].

In addition to the results of the LOS, some improvement was also observed in the postural stability in both the placebo and vitamin D supplemented groups, which indicates the positive effects of surgery and rehabilitation as previously reported [28].

Another important aspect of this study was the timing of the rehabilitation. To the best of the authors’ knowledge, this is one of only a few studies on a supervised rehabilitation program that started as early as four weeks after surgery and allows for safe spontaneous exercises immediately after hospitalization [29].

It is important to note that this study did not observe any adverse effects. Furthermore, our results indicated that early rehabilitation associated with vitamin D intake had beneficial effects with a faster return to balance, recovery, and better postural control after PLIF surgery.

Recently, the authors showed a lower level of lipid and protein peroxidation in the multifidus muscle of patients supplemented for five weeks with vitamin D before PLIF surgery [30]. The muscles perform a fundamental function in stabilization and postural control as well as a role in maintaining the right balance. The possibilities of maintaining proper equilibrium can connect with the somatosensory, visual, and vestibular systems [31]. The vestibular system stabilizes the gaze on the subject during a head movement by the vestibuloocular reflex. In addition, the vestibulospinal reflex is responsible for the maintenance of posture and balance through the regulation of the muscle tone during the rest and movement of the body [32]. The positive influence of vitamin D supplementation during the patients’ recovery process, as observed in this study, may be explained by the presence of vitamin D receptors in the skeletal muscle and brain. Furthermore, the authors recently found a reduction in the concentration of muscle atrophy markers in the paraspinal muscle in LBP patients supplemented with vitamin D [9]. Based on our data and previous report, the authors postulate that vitamin D positively affects the rehabilitation program as it reduces the atrophy of the muscles responsible for spine stabilization in the lumbar section.

The main focus of the rehabilitation program was the performance of systematic exercises, which should improve postural stability. It can be concluded that the rehabilitation objectives were reached because both groups of patients demonstrated some improvements in postural stability, which might have been a consequence of the strengthened tonus of the stabilizing muscles and improved proprioception.

However, additional, supportive measurements, such as muscle strength and power tests, could not be made due to insufficient spine stabilization so early after the surgery. Additionally, the authors did not want to expose them to destabilizing forces in the operated segment, which could lead to subluxation of the segment.

Based on our data and Dzik et al., it is assumed that the better function of the stabilizing muscles resulted in better fusion and faster recovery after PLIF surgery [30].

Most of the studies where the outcomes of rehabilitation were investigated were carried out between three and six months after surgery [4,33]. In this study, the supervised rehabilitation program, which started four weeks after surgery, required patients to make the effort to leave the house, drive a car, or use public transport in order to arrive at the university rehabilitation units. These should be considered as an additional positive aspect of our program as patients were stimulated to be more independent.

## 5. Conclusions

In conclusion, Vitamin D supplementation ameliorated the effects of an early postoperative rehabilitation program implemented four weeks after posterior lumbar interbody fusion. Early physiotherapy treatment after PLIF surgery combined with vitamin D supplementation appears to be a very important combination with regard to the patients’ recovery process.

## Figures and Tables

**Table 1:** The details of the rehabilitation protocol used for the patients.

Week	Intervention
I	Instruction on ergonomic behavior during daily activities; instruction about deep trunk stabilization; and movements of the upper and lower limbs were performed while maintaining a neutral posture.
II–III	The same exercise as above but with increased challenge. Previous exercises were difficult; continuing deep stabilization with dissociated exercises; patients performed exercises in a closed chain at the wall in a standing position, which prepared them to plank, and balance exercises.
IV	Balance exercises with sensorimotor discs.
V	Additional isometric contractions of stabilizing muscles, changes in sequence and duration of movements.

**Table 2:** The impact of vitamin D supplementation on the postural stability test and risk of fall test.

	Vit D Supplemented (EG)		Control Group (CG)		rANOVA			
	Baseline (*n* = 15)	After 5 weeks of supplementation (*n* = 15)	Baseline (*n* = 18)	After 5 weeks of supplementation (*n* = 18)	Time	η_p_^2^	Group x time interaction	η_p_^2^
General	0.35 (0.15)	0.27 * (0.08)	0.34 (0.20)	0.32 (0.18)	*p* = 0.01	0.19	*p* = 0.47	0.02
AP	0.21 (0.07)	0.19 (0.08)	0.32 (0.16)	0.25 * (0.15)	*p* = 0.00	0.25	*p* = 0.04	0.13
ML	0.12 (0.09)	0.11 (0.08)	0.16 (0.09)	0.14 (0.07)	*p* = 0.45	0.02	*p* = 0.88	0.00
RFT	1.03 (0.50)	0.91 (0.38)	1.16 (0.69)	1.01 (0.39)	*p* = 0.20	0.06	*p* = 0.81	0.00
25-0HD [nmol • L^−1^]	48.37 (9.51)	75.10 * (10.25)	51.35 (18.05)	49.46 (11.84)	*p* = 0.00	0.52	*p* = 0.00	0.59

Data are presented as means and standard deviations (SDs). Partial eta squared (ηp^2^) values are provided to estimate the effect sizes of the repeated measures analyses of variance (rANOVAs). Significant differences were set at *p* < 0.05, * = significant differences. PST = postural stability test; OSI = overall stability index; AP = anterior–posterior stability index; ML = medial–lateral stability index; RFT = risk of fall test; 25-OH-D = vitamin D; Pl = placebo.

**Table 3:** Vitamin D supplementation ameliorates the effects of five weeks of rehabilitation of patients after posterior lumbar interbody fusion (PLIF) surgery.

	Vit D Supplemented (EG)		Control Group (CG)		rANOVA			
	After operation (*n* = 15)	After 5 weeks of rehabilitation (*n* = 15)	After operation (*n* = 18)	After 5 weeks of rehabilitation (*n* = 18)	Time	η_p_^2^	Group x time interaction	η_p_^2^
General	0.31 (0.08)	0.23 * (0.08)	0.33 (0.18)	0.29 (0.11)	*p* = 0.02	0.17	*p* = 0.46	0.02
AP	0.26 (0.18)	0.19 * (0.08)	0.24 (0.08)	0.22 (0.09)	*p* = 0.03	0.15	*p* = 0.19	0.05
ML	0.13 (0.07)	0.07 * (0.04)	0.13 (0.08)	0.13 (0.07)	*p* = 0.05	0.14	*p* = 0.04	0.14
Risk	0.91 (0.38)	1.03 (0.38)	1.01 (0.39)	1.08 (0.63)	*p* = 0.12	0.08	*p* = 0.74	0.00
25-0H-D [nmol • L^−1^]	75.10 (10.25)	67.11 (11.45)	49.46 (11.84)	43.62 (11.92)	*p* = 0.00	0.34	*p* = 0.57	0.01

Data are presented as means and standard deviations (SDs). Partial eta squared (ηp^2^) values are provided to estimate the effect sizes of the repeated measures of the analyses of variance (rANOVAs). Significant differences were set at *p* < 0.05, * = significant differences. PST = postural stability test; OSI = overall stability index; AP = anterior-posterior stability index; ML = medial-lateral stability index; RFT = risk of fall test; 25-OH-D = vitamin D; Pl = placebo.

## Abbreviations

PLIF	Posterior Lumbar Interbody Fusion
CRP	C-Reactive Protein
IL-6	Interleukin 6
BBS	The Biodex Balance System
PST	Postural Stability Test
LOS	Limits of Stability Test
RFT	Risk of Fall Test
MRI	magnetic resonance imaging
